# Addressing the inequity of access to home Dialysis in Europe: recommendations for action informed by an international consensus exercise

**DOI:** 10.1186/s12882-025-04188-y

**Published:** 2025-06-03

**Authors:** Simon J. Davies, Bert Bammens, Ilaria de Barbieri, Edwina A. Brown, Jan van Cruchten, Dani Gallego, Eric Goffin, Maurizio Gallieni, Sotiroula Gliki, Jeroen P. Kooman, Gert Meeus, May-Britt Moeslund-Hansen, Peter Rutherford, Raymond Vanholder, Martin Wilkie, Wim Van Biesen

**Affiliations:** 1 School of Medicine, Faculty of Medicine and Health Sciences, Keele University, Keele, UK; 2 Department of Microbiology, Immunology and Transplantation, KU Leuven, Belgium; 3 Department of Nephrology, Dialysis and Renal Transplantation, University Hospitals Leuven, Leuven, Belgium; 4 University of Padova, Padova, Italy; 5 Kidney and Transplant Services, Imperial College Healthcare NHS Trust, London, Spain; 6 European Kidney Patients Federation (EKPF), Madrid, Spain; 7 Department of Nephrology, Université Catholique de Louvain, Cliniques universitaires St Luc, Brussels, Belgium; 8 Department of Biomedical and Clinical Sciences, University of Milano, Milano, Italy; 9 SHSO Limassol and Paphos General Hospital, Associate Educator Nursing Education Sector Cyprus Ministry of Health and University of Nicosia, Milano, Cyprus; 10 Division of Nephrology, Department of Internal Medicine, University Hospital Maastricht, Kortrijk, The Netherlands; 11 Department of Nephrology, Nederlandstalige Belgische Vereniging Voor Nefrologie (NBVN) (President), AZ Groeninge Hospital, Kortrijk, Belgium; 12 Dialysis International, CEO, Helsingør, Denmark; 13 Vice President, Medical Affairs, Vantive, Zurich, Switzerland; 14 European Kidney Health Alliance, Brussels, Belgium; 15 Department of Internal Medicine and Pediatrics, Nephrology Section, Ghent University Hospital, Ghent, Belgium; 16 Sheffield Teaching Hospitals NHS, Sheffield, UK; 17 Renal Division, Gent University Hospital, Gent, Belgium

**Keywords:** Patient empowerment, Modality choice, Training, Home dialysis, Workforce, Peritoneal dialysis, Home haemodialysis

## Abstract

**Background:**

Use of Home Dialysis (referring to both peritoneal and home haemodialysis throughout this study), is under-exploited and highly variable across Europe, and this is the case both within as well as between countries. Several, predominantly modifiable barriers have been described that explain this inequity of access, but as yet no recommendations have been agreed upon as to how to address the problem.

**Methods:**

A multi-disciplinary multi-organisational policy forum representing the key stakeholders was held at the EuroPD meeting in Bruges, November 2023 with the purpose of defining solutions and actions that the wider nephrology community should take to reduce disparities in access to home-based therapies. Three key themes were identified by a steering group prior to the forum: *Dialysis Provider Motivation*,* Patient Empowerment* and *Training and Workforce Issues.* Breakout discussion groups for each theme were asked to prioritise up to three actions per theme. These were further refined by the steering group and developed into proposed actions to be taken forward by the kidney failure community.

**Results:**

112 registrants attended the forum representing patients (5%), doctors, (57%) nurses, (13%) industry (7%) and various other roles (18%). The following actions were proposed: (1) a granular European audit of financial disincentives affecting decisions of policy makers, providers, patients and industry; (2) engaging national professional societies to challenge complacency towards uptake of home-based therapies; (3) stimulate networking to support small, inexperienced centres; (4) extending access to assisted peritoneal dialysis; (5) greater involvement of patients (locally and nationally) at every step, especially for advocacy; (6) empowering patients with transparent information; (7) mandating inclusion of training and exposure to Home Dialysis in national curricula; (8) promotion of career sub-specialists (doctors and nurses) with specific qualification in Home Dialysis; (9) promoting access to high quality training resources.

**Conclusions:**

The kidney failure community can undertake a number of constructive actions to improve equity of access to Home Dialysis. The Policy Forum steering group who are representative of the key stakeholders have committed to taking this programme forward.

## Key learning points

**What is known** There is substantial variability and under-use of Home Dialysis across Europe pointing to inequity of access. Multiple modifiable causes have been identified.

**This study adds** We report on a multi-disciplinary policy forum that has identified a number of actions to address this inequity. These include a Europe-wide granular audit of financial disincentives, transparency of data, recommendations for networking, availability of assisted peritoneal dialysis, speciality curriculum templates for professionals and training opportunities.

**Potential Impact** These actions are expected to empower patients wishing to access Home Dialysis and physicians wanting to build a Home Dialysis service.

## Introduction

The utilisation of Home Dialysis across Europe is underexploited and highly variable [1–3]. The proportion by country of patients initiating treatment for kidney failure with peritoneal dialysis (PD) in 2021 varied between 1% and 30%, (median 13%) [3] and the situation is even worse for home haemodialysis (hHD), which is even not available in some countries [2]. These differences cannot be justified by inferior outcomes as when compared to in-centre HD (cHD), survival is at least equivalent for PD [4–6] and even superior for hHD [7–10]. Furthermore, beyond survival, life participation is an important aspect of modality choice as dialysis is, unfortunately, a ‘way of life’ [11, 12]. Having some control over their treatment has advantages for many, with Home Dialysis allowing individuals to work, travel, spend more time with friends and families and where possible, empower them to live their lives to the best of their ability. These advantages pertain to people of all ages and we know patients care deeply about this as articulated in their own manifestos [13, 14]. The International Home Dialysis Consortium which is addressing the lack of access to Home Dialysis worldwide has also recently published its manifesto: www.ispd.org/IHDC.

**Fig. 1:**
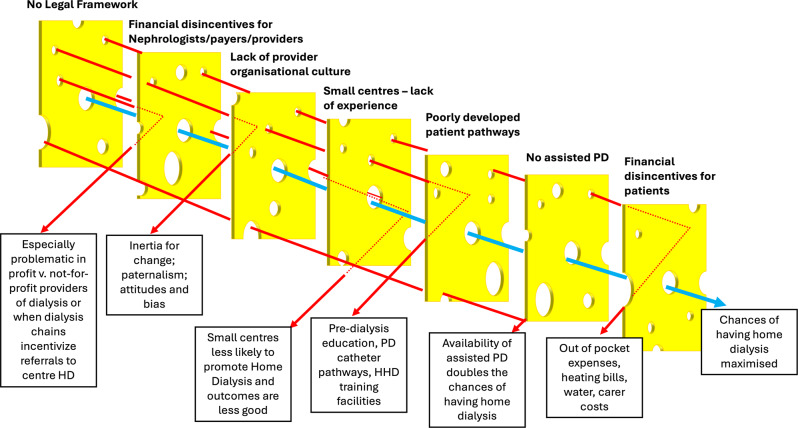
The multiple barriers that affect equity of access to Home Dialysis in Europe can be illustrated using the Swiss Cheese analogy. Each cheese slice is a barrier, arranged hierarchically according to their permissiveness. For example, some European countries do not have a legal framework in place to allow hemodialysis to be undertaken unsupervised at home. Access to Home Dialysis is maximised when the cheese barriers are overcome (as shown by the blue arrow)

Much has been written about the presumed barriers to Home Dialysis that are used as excuses to explain away inequity of access [15–22]. With a deeply rooted perception that in-centre HD is the “default option”, all these barriers act as multiple layers that each need to be overcome for patients to have un-impeded modality choice (Fig. 1). They include financial (dis)incentives; [23] insufficient attention to the ‘patient voice’; organisational culture (including lack of motivation); [24] poor dialysis preparation, including patient education and PD catheter access pathways; [15] a crisis in the European health workforce [25] especially for nurses [26], despite Home Dialysis being paradoxically less nurse intensive than cHD; small centre size; [27] lack of experience and insufficient training opportunities [15]. Achieving equity of access means that some patients who may wish to have treatment at home need extra support: availability of assisted PD and its reimbursement varies dramatically across Europe but when it is available it doubles the number of patients who have treatment at home [28]. Support of assisted home hemodialysis is almost non-existent. Last, but not least, there is world-wide a decline in the interest in pursuing a career in nephrology [29]. 

It might be argued that these barriers may be overcome by the application of financial levers or ‘nudges’, and in some settings this approach certainly plays a role, especially as reimbursement for Home Dialysis is lower than for centre-dialysis in Europe, despite being overall cost saving [30–34]. The ability of payers to influence the use of Home Dialysis is clearly demonstrated in the US [35], but translating the US experience to European countries is difficult [36] as approaches to healthcare funding are different and this is not an area in which the European Union can dictate policy. It is thus very unlikely that a Europe-wide, top-down policy to stimulate home-based dialysis therapies will emerge spontaneously without considerable efforts from the nephrology community.

Many, arguably all, of these barriers to home based therapies fall under the direct responsibility of healthcare professionals, who in some circumstances, due to conservatism may act as gate-keepers preventing the growth of Home Dialysis rather than using their expertise to influence policy makers. With this in mind, and to stimulate action, *EuroPD*, a not-for-profit organisation that supports the nephrology community in delivering best quality peritoneal and home haemodialysis, sponsored a Policy Forum at its international meeting held in November 2023 entitled *Addressing the Inequity of Access to Home Dialysis in Europe*. The objective was to agree upon a discrete number of actions that would form the basis of a roadmap for the wider nephrology community to foster an ethical response to the inequities in Home Dialysis uptake.

## Materials and methods

### People and organisations

It was important to involve representation from the key stakeholders in this process, so the Forum was planned in collaboration with leadership from the following organisations: *European Kidney Health Alliance*, *European Kidney Patients’ Federation*, *International Society for Peritoneal Dialysis* (including representation from the *International Home Dialysis Consortium* that is co-hosted by the *International Society of Nephrology*), *European Dialysis and Transplant Nurses Association – European Renal Care Association*, *Fresenius Medical Care and Baxter HealthCare*. The *European Renal Association* was invited but did not have formal representation at the Forum, although colleagues involved with developing their training materials were invited individually. A steering group of members of these organisations was established and through online meetings planned the objectives and format of the forum.

### Format and process

**Fig. 2:**
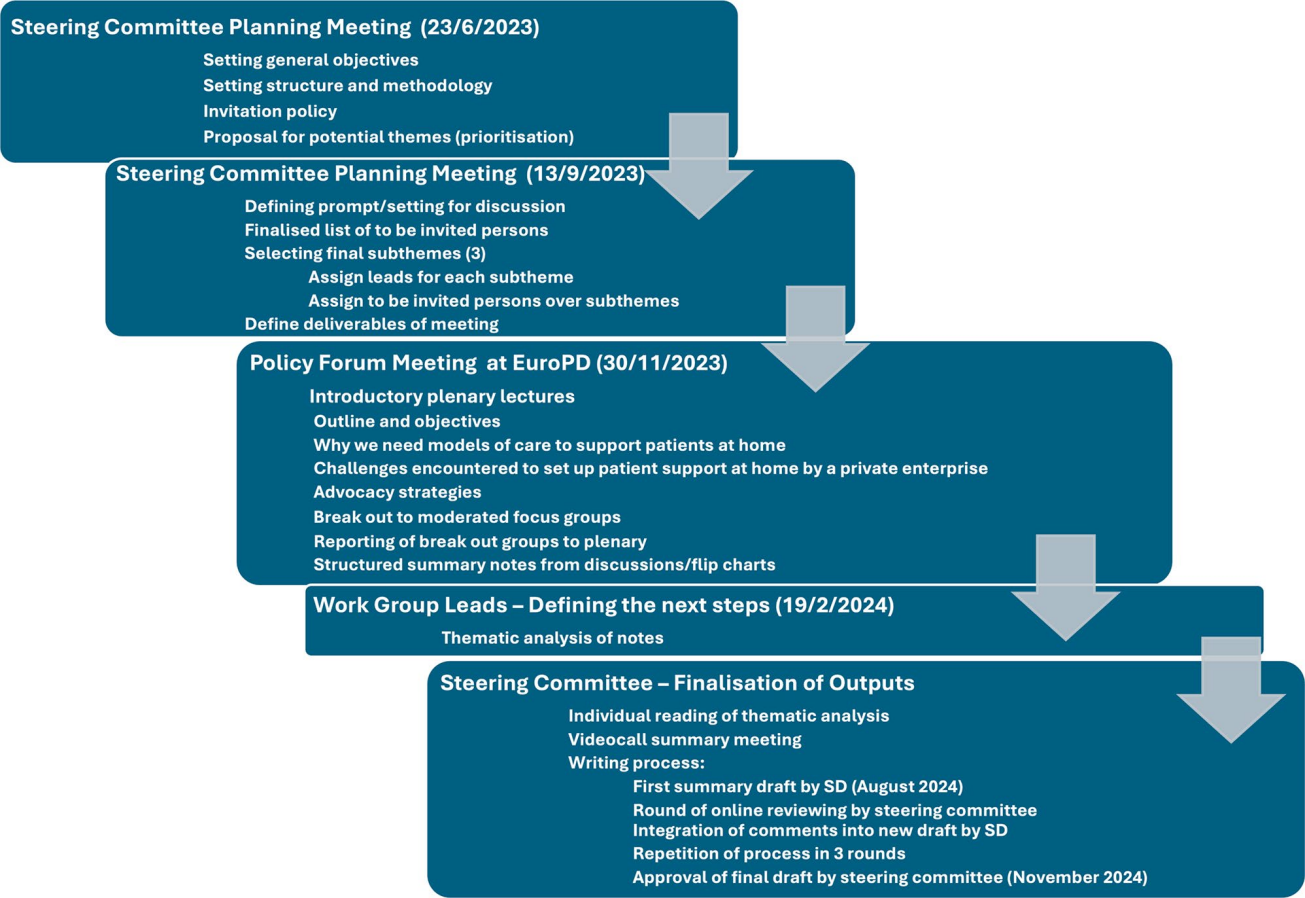
Diagram summarising the process of developing the policy forum and formulating the recommendations and action plan

This is summarised in Fig. 2. The steering group decided that the primary target for recommendations should be professionals involved in kidney failure care, in particular professional bodies such as national and international societies, rather than governmental bodies or the European Commission. It was agreed that creating specific recommendations tailored to different healthcare systems would be too ambitious. It was recognised that additional specific invitations would need to be made to ensure adequate representation from stakeholders, in particular patients and their families or carers, and that their participation would need to be funded to ensure equity and independence. The overall structure of the Forum is shown in Table 1. The breakout groups were asked to propose and prioritise solutions and recommendations, but not be constrained by funding considerations.

The scope of the three breakout themes were: (1) *Dialysis Provider Motivation* (2), *Empowering the Patient* and (3) *Education and Workforce Issues* as shown in Table 1. Each discussion area was assigned moderators to capture key-points of the discussion on flipcharts, not only to document and summarize, but also promote feedback and further flesh out the essential elements. This was followed by a moderated plenary discussion of each of the themes.

After the meeting, notes from this discussion and the breakout meetings were elaborated further by the moderators of each subgroup separately and summarized versions discussed by the whole steering group during a structured online meeting in February 2024. The weighting and evaluation of each of the themes was finally distilled into the key recommendations. At none of the decision points were votes taken as the conclusions were always based on consensus. A structured thematic analysis was performed to distil out clear and distinctive concepts, aiming to understand and correctly formulate the fundamental basis of barriers and proposed solutions.

**Table 1:** Format of the policy forum including plenary presentations, main themes and sub-topics to be covered by breakout groups

Plenary Session	
Outline of the Policy Forum objectives and the format, including a brief summary of the current status of Home Dialysis in Europe and known challenges	Simon Davies *(President of EuroPD and Policy Forum Chair)*
Why we need models of care that support patients at home	Jan van Cruchten *(Secretary of the European Kidney Patients Federation)*
Challenges to setting up services to support Home Dialysis in Belgium	Conversation between Caydie Van Brabant *(Hayhealth*) and Wim van Biesen *(Vice-chair EuroPD)*
How to do Advocacy	Raymond Vanholder *(President of the European Kidney Health Alliance)*
**Breakout Session Theme Headings**	**Range of Topics to be covered**
1. Dialysis Provider Motivation	Re-imbursement and financial incentives, Attitudes and bias, organizational culture and lack of motivation. Effect of centre size and provision of assisted PD
2. Empowering the Patient	How can kidney failure patients and their families be empowered? What determines the effectiveness of the ‘patient voice’? Impact of poor dialysis preparation that excludes Home Dialysis, with negative attitudes and biases towards patient empowerment
3. Education of Professionals and Lack of Staffing	How to solve the staffing crisis– what is needed? Is Home Dialysis a solution? What is needed to develop careers in Home Dialysis? What is required to facilitate professional development in Home Dialysis?

## Results

The Policy Forum was attended by 112 pre-registered participants from 22 European countries, with representation from patient groups (including family members, 5%), nephrologists (including trainees, 57%), nurses (13%), industry (7%) and other roles including managers, engineers, project managers, educators and a psychologist (18%). Given the nature of the conference, there was an inevitable bias towards countries that support Home Dialysis well, but there was also representation from countries with low penetration (e.g. Albania, Croatia, Cyprus, Greece, Poland, Slovenia).

### Dialysis provider motivation

Three major themes, along with their potential solutions and proposed actions emerged from these discussions, albeit with considerable overlap: *(1) financial incentives; (2) culture of care*,* networking and collaboration; (3) provision of assisted PD*.

#### Financial incentives

It was recognised that healthcare systems where financial incentives are disadvantageous to Home Dialysis definitely impede its use. A level financial playing field is essential to ensure equity of access to Home Dialysis. However, it was recognised that the way money flows may vary considerably by country, and that it is complex and difficult to define, with sometimes hidden or unintended consequences of financial incentives, however well intentioned. The group agreed that more transparency in the form of a granular audit at an individual European country level was needed, allowing determination of the exact financial flows. As specified by the KDIGO controversies conference [10] this audit should include the four main economic drivers determining the use of Home Dialysis: The Funder/Payer (policy favouring/disfavouring Home Dialysis), the Dialysis Provider (profit/not-for-profit), the Nephrologist (source of income/reimbursement, salary/fees/shared ownership, unofficial incentives), and the Patient (out of pocket expenses, employment, travel). The results of such an audit should be published and thus be made available as a resource, likely in the form of a directory, to policy makers, clinicians and patient groups (see Table 2).

**Table 2:** Summary of prioritized recommendations and actions

Recommendations	Actions	Comments
**Theme 1: Dialysis Provider Motivation**		
Establish how the money flows in order to see what is required and where to ‘level the playing field’	Undertake a granular audit across Europe of the economic drivers as they affect funders, providers, nephrologists and patients	Publish results; EuroPD will support this activity
Establish a culture of PCC as an ethical imperative in the preparation and the application of Home Dialysis	Audit the approach national societies are taking to ensure professionals are encouraged and able to practice PCC	Engage national/ international societies
Develop solutions for the ‘small centre’ problem	Promote networking as a model of professional organisation	Publish successful examples
Promote universal access to assisted PD in Europe	Work with professional societies to define the country level barriers to assisted PD	Include the use of assisted PD in ERA Registry Reports
**Theme 2: Empowering Patients**		
Involve patients at every level – local (service organisation) and nationally	Establish the ability of national KPAs to engage with professional bodies and policy makers and how they might be supported	Collaborative project with EKPF and EKHA. Links to PCC above
Make transparent information available, including availability of treatments, their use and associated outcomes	Audit the approach of professional societies in ensuring patients are able to access information, e.g. accessible comparisons of unit’s outcomes and patient experience of care	Role for ERA Registry to enhance reporting on Home Dialysis providing more detail
Education to the appropriate level of patients, professionals, trainees, students	This requirement should inform the actions of Theme 2 at every level.	See below
**Theme 3: Workforce and Education**		
Promote sub-speciality career development for nurses in Home Dialysis	Develop a European Nurses Home Dialysis curriculum template, that includes ‘train the trainer’ and ‘train the patient/adult learner’ skills and patient empowerment/advocacy skills	Collaborative project led by EDTNA-ERCA
Promote sub-speciality career development for nephrologists in Home Dialysis	Audit inclusion of Home Dialysis in national nephrology training curricula	Publish findings
Underpin these recommendations by curriculum development and training opportunities with the aim of mandating Home Dialysis training and experience in national and European curricula.	Publicise existing training tools and materials (e.g. ERA Neph-Manuel, NPATH, ISPD) and ensure that they have Home Dialysis content, e.g. service management and leadership skills and PCC skills, e.g. promoting shared decision making and voiding unconscious bias	Collaborative project with providers of Home Therapies Education (ERA, ISPD, EuroPD, EDTNA-ERCA and NPATH)

PCC: Patient/Person-centred care; KPA: Kidney Patient Associations; EKPF European Kidney Patient Federation; EKHA European Kidney Health Association; ERA: European Renal Association; EDTNA-ERCA: European Dialysis and Transplantation Nurses Association – European Renal Care Association

#### Culture of care and networking

Although this group recognised that removing financial disincentives is a *necessary* condition it is not *sufficient* in ensuring equity of access to Home Dialysis. The problem of dialysis provider inertia was identified with several accompanying facets, including attitudes, often historically negative but now inaccurate, towards Home Dialysis, an unwillingness to refer and send patients to other colleagues with the appropriate expertise, paternalism and a lack of ‘patient centeredness’. Collectively these characterise the dialysis provider’s organisational culture. Conversely, a positive culture is characterised by a shared belief in Home Dialysis as a desirable treatment for a significant proportion of patients with a presumption of their eligibility for this modality and provision of support where this is needed [24]. It is recognized that the proportional uptake of Home Dialysis may largely differ by centre, region or country. Some of this variation is to be expected but as indicated in the introduction a substantial amount is unwarranted.

Creating the optimal culture of care is about acting in the patient’s best interests, incorporating best practice (for example embedding shared decision making) and as such is a matter of professional ethics. It was felt that national and international professional societies have not always taken sufficient responsibility to actively work on these themes. There was an impression that the content of educational initiatives and conferences in the field of nephrology are often largely driven by the interests of industry, academia or dialysis providers, rather than by what is truly important to patients. An audit of how professional organisations across Europe have made steps towards patient inclusiveness with respect to Home Dialysis is proposed as an action point (see Table 2).

Networking was seen as a potential solution to some of these issues, especially given the large number of small centres in Europe, or centres with very small percentages of their patients on Home Dialysis. Ideally, such networks should not be financially competitive, share expertise, develop centres of excellence within their cluster and be incentivised for their collaboration. Paediatric practice (where centres are usually small and collaboration is encouraged) as well as existing incentives applied in some countries (e.g. professional networks in Norway or the structure of the Spanish transplantation model) were seen as good examples of how networking can be incentivised and bring added value.

#### Assisted PD

The evidence that assisted PD improves equity of access to PD for particular subgroups of patients is unequivocal. It enables principally older, frail patients to have the choice of receiving their dialysis at home, avoiding the hardships of transport, including the associated costs, as well as prolonged post-haemodialysis recovery. Failure of provision is, in effect, discriminatory towards this expanding group of patients. However, there are important barriers to overcome which are not only financial, although additional costs need to be reimbursed. In some countries there is a need for change in legislation to enable nurses and in particular healthcare assistants to deliver dialysis at home. There is again a role for professional societies to prioritise and advocate for change, perhaps in collaboration with other medical specialities (e.g. geriatric services) in delivering complex medical care in the home setting.

### Empowerment of patients

Several inter-related themes emerged during the discussions: the *patient as advocate* for Home Dialysis, the importance of respect for *autonomy*,* transparency of information*, including *shared decision making* and the imperative to *involve patients* in all aspects of service development and delivery, peer support, taking an active role in their own care, including learning to undertake treatment related tasks [37].

Listening to patient testimonies it was clear that patients are the best advocates for Home Dialysis. They have both a local and a national/international role in promoting and educating fellow patients and professionals about Home Dialysis and challenging its lack of provision. Professionals and policy makers need to empower patients in these roles, which might be done at the local level within centres by involving them in patient education and service design, whereas nationally there is a need for strong patient organisations in every country. Empowerment training initiatives like EUPATI, https://eupati.eu, can provide patient organisations and professionals with tools to achieve this.

Provision of unbiased, *transparent information* is central to the empowerment of patients, their families and carers. A view strongly expressed was that provision of such information should be an official, documented and verifiable requirement, whereas the concept of mandating targets for proportions of patients being treated with Home Dialysis was less well supported. Patients expressed the concern that there were ‘attitudinal’ barriers when it came to being listened to in an unprejudiced way. They preported experiencing that clinicians often had their own agenda, and that on many occasions, potential candidates for home based therapies were being dissuaded to pursue Home Dialysis by in-centre HD staff or medical professionals not directly involved in their kidney care. It was recognised that this often reflected of lack of education and experience of professionals not experienced in home based therapies. This underlines the importance of educating the whole workforce, highlighting the need for a common vision and culture within the nephrology unit, and even the larger health care organisation, on the importance of patient empowerment, shared decision making and promotion of Home Dialysis therapies [38]. 

### Workforce shortage and professional education

Although these issues are interlinked they were discussed separately in the breakout groups. It was generally acknowledged that there is a *workforce crisis* in European medical care, in particular in nursing, and that increasing Home Dialysis use is, at least in theory, a solution for this problem. However, in practice the reverse is usually the case as the crisis of staffing in the inpatient and in-center HD services frequently leads to nurses being withdrawn from the Home Dialysis team, so perpetuating a vicious cycle that precludes development of Home Dialysis services. This is further exacerbated by lack of policies and funding that enable nurses to work in the community.

#### Incentivising nurses

In all of the renal nursing sub-specialities there is a need for change. Nurses report feeling under-valued compared to other specialities, expressing that there is a need to change this attitude so that their contribution is recognized and valued. There is a need to develop clearer career pathways in which nurses are properly remunerated for the responsibilities and expertise they have. In particular, with respect to Home Dialysis, they wish to undertake more responsibility, developing and delivering patient-centred care, but rightly feel that this is currently undervalued. For example, nurses specialising in intensive care or midwifery have better employment conditions, are recognised for their skills and are often paid more. Solutions discussed included the need to develop roles for ‘dialysis assistants’ to support fully trained and experienced nurses, especially in the delivery of assisted PD and hHD. It is recognised that in some countries there are legal barriers to nephrology nurses undertaking treatments in the home and that this will require careful examination. Furthermore the development of a Europe-wide standardized curriculum template would help establish Home Dialysis as a recognised nursing sub-speciality.

#### Education of nurses

In parallel with the need for career pathways that encourage nurses to engage in Home Dialysis is the need to better develop sub-speciality training. This also applies to nurses working in the pre-dialysis clinic and should include expertise in training adults (including training the trainers). Training should be structured around the proposed professional curriculum and be evidence-based. Utilizing existing ISPD and EDTNA/ERCA accredited guidelines and teaching materials can be a good starting point, although these will often require translation and adaptation to local circumstances. Nurses should also be encouraged and supported by their employers to do research and engage in quality improvement.

#### Training and educating nephrologists

Although the workforce crisis in nursing is critical, there is also a challenge in many countries for nephrologists, which will especially impact on those with an interest in home based therapies. Incentivizing nephrologists to devote time to Home Dialysis is clearly linked to the need for ensuring that this is not to their financial disadvantage as discussed earlier. Training of nephrologists also requires a professionally endorsed training curriculum. Not all European nations have a nephrology curriculum that covers Home Dialysis. Developing a Europe-wide curriculum has been considered too complex by the ERA, which instead is developing a “Neph-Manual” initiative to support nephrology training across the board (neph-manual@era-online.org). There is a Europe-wide examination available, ESENeph, (https://www.thefederation.uk/examinations/european-specialty-examination-nephrology) which does include peritoneal dialysis, but this is not required for registration so very few nephrologists take it. Training and certification in home dialysis should be a mandated component of both national and European curricula. This is challenging given the fact that curricula between countries are not yet harmonized. Until this is achieved a national and international network of training centers offering common educational initiatives on home dialysis could overcome the lack of experience. These initiatives should further be underpinned by approved focused training programmes, for example the recently completed European NPATH project, (*npath.eu/login)* as well as other existing resources, e.g. those available at the website of the International Society for Peritoneal Dialysis (https://ispd.org/guidelines/). Next steps are to establish the extent to which Home Dialysis is included in all national curricula across Europe and provide help in developing these alongside Home Dialysis teaching courses such as exist in many countries. An essential component of curricula and training is the inclusion of skills required to run and manage a Home Dialysis program. The role of *networking* in supporting both nephrologists and nurses to gain more experience in Home Dialysis as discussed above was also emphasised.

These discussions, along with the steps required to implement change in Europe, are further summarised in Table 2. The various groups represented at the forum are committed to working together to achieve these objectives and the expectation is to review progress in these projects over the next two years.

## Discussion

This synthesis of the Policy Forum held at the Brugge meeting of EuroPD in November 2023 documents the first collaborative effort to engage with the inequity of access to Home Dialysis in Europe and develop a multi-professional plan for change. The unsatisfactory uptake of Home Dialysis is a deep-seated problem which has been recognised for many years and is the consequence of many factors. However, rather than looking backwards and seeing these well-established barriers as insurmountable, the consensus was to focus on what is new and emphasise a positive viewpoint towards change.

First, the genuine concern of previous generations that Home Dialysis puts patients at risk cannot any longer be supported; second, the ‘paternalistic’ approach in the practice of medicine is gradually being eroded – patients and their families rightly expect this to change and for a treatment that has such a huge impact on how they live their lives it is essential that they are in the driving seat [39]. Third, there is both a financial cost and human resource crisis in medicine. Health economic analyses have repeatedly shown that Home Dialysis is cost effective in Europe and there are clear workforce benefits to keeping patients out of hospital and dialysis units [30–32]. 

Nevertheless, achieving change, especially when many of the players have a vested interest in the *status quo*, is notoriously difficult. The forum unanimously identified that financial incentives are the key determinant of Home Dialysis use and that removal of financial disincentives is an essential and necessary step in achieving change. In shining a light on how this varies across Europe by conducting a granular audit of this complex issue, we will give advocates for change the tools with which to influence policy makers.

It is clear that changing financial incentives, although necessary, is not the only solution to address this challenge. In many units, there will be a need for a change in organisational culture. For example, certain clinicians are unwilling to promote Home Dialysis as they allegedly want to preserve ‘continuity of care’, which in fact means that they are reluctant to refer their patients to another specialist or centre while they themselves lack the expertise or service infrastructure. This is a clear example of putting their own interests before those of their patients. Organisational culture is usually defined as the collection of values, expectations and practices that guide and inform the actions of all team members. Fundamental to this is the shared belief in the practice of patient-centred medicine and the conviction that for many people Home Dialysis is the best option. As identified by the Inter-CEPt study, this can only be achieved by shared decision making, recognition of unconscious bias, presumption of eligibility (rather than searching for reasons why patients are not candidates for home based therapies) and the provision of appropriate support where this is needed [24, 40]. It is recognised that this requires effort, but it must be questioned why there appears to be so much inertia in developing an organisational culture that supports Home Dialysis. It is for this reason that the authors have framed the recommendation to practice patient-centred care as an ethical imperative.

It will be noticed that many of the actions we have proposed require a dialogue with professional bodies which surely have a role in supporting high ethical standards in practice. A better understanding of how national professional bodies support nurses and doctors in this, and in particular how open they are to welcoming patient representatives into their organisation and how they support patients in influencing policy makers will be important to ascertain. Equally there is clearly an important role for the enhanced publication of metrics related to Home Dialysis. Transparent data empowers patients and clinicians who wish to drive change.

Finally, for both nurses and doctors the goal should be that Home Dialysis is a recognized subspeciality of kidney care. Our focus here has been on the development of curricula after establishing whether individual countries have already incorporated appropriate content into existing training programmes. Developing template curricula and collaborating with existing partners to provide accessible and practical training materials will be essential.

This synthesis of recommendations to increase equity of access to home dialysis in Europe has a number of limitations. As already acknowledged, representation at the Policy Forum was biased towards those already in favour of Home Dialysis despite the fact that there were delegates present from countries in which penetration is low. Similarly, members of the steering group were largely, but not exclusively drawn from those advocating Home Dialysis. This point only serves to emphasise that fact that Home Dialysis provision is too dependent on the enthusiasm of clinicians and their teams and this underlines the imperative to the engage the nephrology community in the ways we have proposed in the summary recommendations.

## Data Availability

Data sharing is not applicable to this article as no datasets were generated or analysed during the current study.
